# Differential Effects of Comorbid Psychiatric Disorders on Treatment Outcome in Posttraumatic Stress Disorder from Childhood Trauma

**DOI:** 10.3390/jcm10163708

**Published:** 2021-08-20

**Authors:** Nele Assmann, Eva Fassbinder, Anja Schaich, Christopher W. Lee, Katrina Boterhoven de Haan, Marleen Rijkeboer, Arnoud Arntz

**Affiliations:** 1 Department of Psychiatry and Psychotherapy, Lübeck University, 23538 Lübeck, Germany; eva.fassbinder@uksh.de (E.F.); anja.schaich@uksh.de (A.S.); 2 Department of Psychiatry and Psychotherapy, Christian-Albrechts-University Kiel, 24105 Kiel, Germany; 3 Faculty of Health and Medical Sciences, University of Western Australia, Perth, WA 6009, Australia; chris.lee@uwa.edu.au (C.W.L.); katrina.boterhovendehaan@research.uwa.edu.au (K.B.d.H.); 4 Department of Clinical Psychological Science, Faculty of Psychology and Neuroscience, Maastricht University, 6200 MD Maastricht, The Netherlands; marleen.rijkeboer@maastrichtuniversity.nl; 5 Department of Clinical Psychology, University of Amsterdam, 1000 GG Amsterdam, The Netherlands; A.R.Arntz@uva.nl

**Keywords:** posttraumatic stress disorder, depression, anxiety disorder, therapy, prediction, moderator, treatment outcome

## Abstract

Patients with posttraumatic stress disorder (PTSD) frequently have comorbid diagnoses such as major depressive disorder (MDD) and anxiety disorders (AD). Studies into the impact of these comorbidities on the outcome of PTSD treatment have yielded mixed results. The different treatments investigated in these studies might explain the varied outcome. The purpose of this study was to examine the impact of these comorbidities on the outcome of two specific PTSD treatments. MDD and AD were analyzed as predictors and moderators in a trial comparing 12 sessions of either eye movement desensitization and reprocessing (EMDR) or imagery rescripting (IR) in 155 adult patients with PTSD from childhood trauma. The primary outcome was reduction of PTSD symptoms (clinician-administered PTSD Scale for DSM-5, CAPS-5) assessed at eight-week follow-up and a secondary outcome was self-report PTSD symptoms (Impact of Event Scale, IES-R). MDD was not a predictor of treatment outcome but did have a significant moderator effect. Patients with MDD showed a better outcome if they were treated with IR, whereas patients without MDD improved more in the EMDR condition. No impact of AD emerged. It seems essential to consider comorbid MDD when planning PTSD treatment to improve treatment outcomes. More research is needed to replicate our findings and focus on different kinds of PTSD treatments and other comorbidities.

## 1. Introduction

Patients with posttraumatic stress disorder (PTSD) show high rates of comorbidity with other psychiatric disorders, especially major depressive disorder (MDD), anxiety disorders (AD), and substance use disorders (SUD). These findings are well established in various PTSD populations such as veterans [1,2], survivors of natural disasters [3,4,5], refugees [6], survivors of (interpersonal) violence and rape [7,8], and adults with childhood trauma [9,10,11].

Several epidemiological studies indicate that comorbid MDD, AD, or SUD are related to higher symptom severity [9,12], and lower remission rates of PTSD symptoms [5,13,14,15]. In psychotherapy treatment outcome studies investigating the impact of comorbid disorders on treatment outcome in PTSD symptoms show inconsistent results. The presence of a comorbid depressive disorder predicted a poorer PTSD treatment response in a study of patients with a road traffic collision treated with cognitive–behavioral therapy [16] and in a sample of refugees treated with either eye movement desensitization and reprocessing (EMDR) or stabilization [17]. One study of prolonged exposure treatment for PTSD has found higher dropout rates for patients with comorbid depression [18]. In another study of prolonged exposure in patients with chronic PTSD, depression, and anxiety disorders were not related to either treatment outcome or dropout, but a negative impact of benzodiazepine and alcohol use was found [19]. A study comparing EMDR with a combination of imaginal exposure and cognitive restructuring also did not find a predicting effect of either depressive or anxiety symptoms on treatment outcome [20]. Finally, in traumatized women treated with either cognitive processing therapy or exposure therapy, a higher level of depression was even associated with a better treatment outcome in both conditions, but there was no differential effect between the two treatments [8]. Two recent systematic reviews aimed to identify trajectories and predictors of psychotherapeutic response in adults with PTSD [21,22]: in the first review comorbid depression, anxiety, and alcohol abuse were the strongest predictors of poor therapeutic response [21], while the other only found that comorbid diagnosis of depression was associated with poor treatment outcome, however only two studies [17,23] were included [22].

The variability of the results of these studies might be due to differences in types of traumas, chronicity of the patients, the different comorbidity measures (partly only assessed with self-report measures), and types of psychotherapy—i.e., cognitive behavioral therapy, cognitive processing therapy, EMDR, exposure, etc. Different psychotherapeutic methods might target different underlying mechanisms to reduce PTSD symptoms [24] and comorbid disorders such as depression or anxiety disorders might interfere with these mechanisms in some ways. Also, PTSD patients with specific comorbid disorders might have specific needs that can be better addressed with one method more than another. Hence, it is crucial to investigate specific treatments for PTSD to make more differentiated statements on comorbidities’ impact on treatment outcomes.

Two psychotherapeutic methods with different mechanisms proposed to reduce PTSD symptoms are EMDR and Imagery Rescripting IR [25,26,27]. Both share similarities with exposure techniques such as recalling the trauma memory with related images, emotions, and cognitions, and providing corrective information. However, both limit actual exposure to trauma memories and do not require intensive and prolonged reliving of the trauma [26]. Although each treatment involves processing traumatic memories, different underlying working mechanisms have been proposed [26] and the two approaches are very different in practice. During IR, patients are instructed to imagine the traumatic event as their child self, in a second step the course of the event is changed by imagining a positive outcome with the focus on meeting the patients’ core unmet needs. IR patients do not have to relive the whole trauma in detail as exposure with habituation and extinction is not the primary goal. The predominant explanation for the underlying mechanism is that IR works by facilitating change in the original meaning of the trauma, which results in changes in patients’ core belief systems and behaviors [25,28]. During EMDR, patients recall their trauma experience in brief sequential doses while simultaneously focusing on an external bilateral stimulus such as therapist directed lateral eye movements or hand-tapping. This dual attention focus facilitates the accessing and processing of traumatic memories resulting in a reduction of distress and vividness associated with trauma memories as well as a reformulation of negative beliefs [27]. The precise working mechanism of EMDR is unknown, although there have been several theories proposed [29]. A recent, empirically supported theory of the underlying mechanism of EMDR postulates that eye movements create a high load on the working memory and reduce the vividness and emotional distress linked with trauma memory [30].Whereas EMDR is a common treatment for PTSD, IR has been used less frequently as a stand-alone treatment so far. However, in a recent study comparing EMDR with IR in adults with PTSD from childhood trauma (Ch-PTSD), IR was as effective as EMDR. Both treatments showed large effect sizes from pre-treatment to eight week follow-up, *d*_IR_ = 1.72, and *d*_EMDR_ = 1.73 (IREM trial) [11]. The purpose of this study was to examine comorbidities in PTSD as (1) predictors of treatment outcome and (2) potential moderators with a differential impact on EMDR and IR using data from the IREM trial [11]. MDD and AD were included in the analysis. We did not investigate SUD in our analyses as substance dependency was an exclusion criterion for the study [11,26]. Only a few cases with substance abuse were present, so the overall number of SUD patients was too small.

We hypothesized that comorbid MDD and AD would be associated with worse outcomes in terms of reduction in PTSD symptoms for each condition. Although previous studies on AD and MDD produced inconsistent results, recent reviews found these two diagnoses among the strongest predictors of poor therapeutic response [21,22]. Moreover, lower remission rates of PTSD symptoms in patients with MDD and AD were observed in epidemiological studies [5,13,14,15].

Given that this was the first study to examine a differential effect of comorbid AD and MDD on treatment outcome in EMDR and IR, we had no specific hypotheses about the potentially moderating effect. This hypothesis was therefore exploratory. Different underlying mechanisms assume there might be a differential effect of these comorbidities in the treatment outcome.

Enhancing knowledge of the impact of comorbid disorders on PTSD treatment is crucial given the substantial comorbidity between PTSD and other psychiatric disorders. It will help to identify those who have the best chance to benefit from treatment, support the choice of a specific treatment in case of differential effects and it is essential to develop modifications of existing treatments or new approaches to better address the needs for those with poor outcome.

## 2. Materials and Methods

This study was based on an international multicenter randomized clinical trial (IREM RCT) comparing two types of psychotherapy for Ch-PTSD, namely EMDR and IR, in seven sites in Australia, Germany, and the Netherlands [11]. The trial was registered on the Australian and New Zealand Clinical Trials Registry (ref no. ACTRN12614000750684). Local institutional review board approval was obtained, and all patients provided written informed consent. The following is a brief description of study characteristics; further information is contained in the IREM design article [26].

### 2.1. Participants

The main inclusion criterion was a current primary diagnosis of PTSD based on an index trauma before the age of 16 with symptoms for at least three months. Participants had to be aged between 18 and 65 years, be able to attend sessions twice a week, and agree to stay on a stable medication (or no medication) during the treatment phase of six-week and the eight-week follow-up phase. Participants had to be excluded if they had (1) an acute suicide risk, (2) a comorbid psychotic disorder, (3) a bipolar disorder type 1, (4) an alcohol or drug dependence, (5) a PTSD from trauma occurring within the past six months, (7) an IQ below 80, (8) medication changes or any PTSD-focused therapy within the past three months, and (9) were on benzodiazepine medication, however, participation was possible after two weeks of abstinence of this medication.

### 2.2. Randomization and Masking

Participants were randomized either to EMDR or IR after pre-treatment assessment using block randomization (*n* = two, four, and six per block, with block size randomized) and stratifying for gender to control distribution per treatment at each site. An error did occur in the randomization only for the first two sites resulting in an early disproportionate, but still random, allocation to EMDR.

### 2.3. Procedures

Participants were recruited at seven mental health and specialized services across Australia, Germany, and the Netherlands from October 2014 to June 2019. Potential participants were screened for psychiatric disorders using the Structured Clinical Interviews for DSM-IV-TR [31] or the Mini International Neuropsychiatric Interview [32], depending on site preference. Diagnostic interviews were based on the DSM-IV classification system because the Structured Clinical Interview for DSM-5 [33] was not available in all languages at the beginning of the study. Trauma history was assessed using the Life Events Checklist for DSM-5 [34]. All assessments were conducted by trained research assistants blind to treatment conditions.

Outcome assessments were conducted pre-treatment, post-treatment, eight weeks post-treatment (follow-up 1), and one year after the pre-treatment assessment (follow-up 2). An additional wait-list assessment was conducted if participants had to wait three weeks or more before the treatment started.

### 2.4. Outcomes

The primary outcome was the change in PTSD symptom severity from pre-treatment to follow-up 1 assessment measured by the clinician-administered PTSD Scale for DSM-5 (CAPS-5) [35]. The CAPS-5 is a well validated semi-structured diagnostic interview to assess severity of PTSD symptoms over the previous month [35]. It consists of 30-items, corresponding to the DSM-5 PTSD symptoms and rates the severity of PTSD within a range of 0–80 (higher scores reflecting greater severity) over the last month. 

The Impact of Events Scale-Revised (IES-R) [36] was used as a secondary outcome for a broad perspective of PTSD symptoms. The IES-R is a 22-item self-report questionnaire measuring PTSD symptoms over the last seven days. Since symptoms are related to a specific traumatic event in the IES-R, participants rated each symptom twice, concerning the index trauma and concerning all other traumas except the index trauma.

### 2.5. Treatment

Treatment consisted of twelve 90-min sessions IR or EMDR, twice a week, for a period of six weeks with up to eight weeks permitted. It was possible to conclude treatment in less than twelve sessions provided that the participant, therapist, and site coordinator agreed the participant had recovered. Both treatment conditions followed standardized treatment manuals [25,27], and all sessions were either video- or audiotaped. 

In IR, the traumatic experiences are addressed by imagining a new script of the traumatic scene. For example, the abuse is stopped, and the child’s needs are taken care of by a helping person. For the first six sessions, the therapist enters the image to help the child. From session seven onwards, the participant imagines himself as an adult helping the child [25]. In the EDMR condition, the eight-phase EMDR protocol developed by Shapiro was followed [27]. After a procedural preparation and an affect tolerance training in session one, from session two on in each session a target memory for processing was selected and EMDR processing was performed according to the Shapiro protocol. In the last session, if not done before, a target situation involving a future situation was selected and processed to overcome residual anticipatory anxiety or avoidant behavior. A detailed description of the psychotherapeutic interventions can also be found in the study protocol [26].

Study therapists were licensed psychologists, psychotherapists, psychiatrists, and one psychiatric nurse with advanced mental health qualifications trained in one or both treatment conditions. For details on training, supervision, and adherence, see also the study protocol and manuscript on clinical effectiveness from the IREM trial [11,26].

### 2.6. Statistical Analysis

Statistical analysis was done with SPSS, version 27 (IBM Corp Armonk, USA, 2017), predefined in the study protocol [26], and used in the analysis to compare the clinical effectiveness of the two methods [11]. In line with the main article, we choose for a multilevel model as this can accommodate site effects, whilst neglecting them would lead to less valid estimations and hence less validity of generalization of conclusions to mental health care in general. Another reason to choose for a multilevel analysis is that this method uses all available data (intent-to-treat principle) and leads to valid conclusions assuming that missing values are random above to what the independent variables in the model predict. This approach is generally viewed as more valid than (a) analyzing only the participants with complete data, (b) single value imputation, and (c) last observation carried forward. Lastly, as the distributions of dependent variables were extremely skewed at later assessments, due to strong effects of treatments (many zero scores), an analysis assuming normal distributions would be invalid. We therefore choose for a generalized linear mixed model based on a negative binomial distribution, sui IREM sample for inherently skewed distributions with discrete values. The hypotheses regarding effects of comorbid AD and MDD on reduction of PTSD symptoms were tested by performing analysis on the whole database including all randomized patients (intention-to-treat analysis). Therefore, diagnosis of MDD or AD, as well as their interactions with time (categorical, pre-treatment as reference) and treatment condition, were added to the GLMM as fixed factors. Since lower-level interactions and main effects cannot be interpreted in a model including a three-way interaction with noncentered predictors, the GLMM was repeated with centered dummies as predictors. After Bonferroni adjustment for multiple testing (MDD and AD), *p*-values < 0.025 were considered statistically significant. To strengthen the findings, we conducted a sensitivity analysis. Therefore, we repeated the primary analyses on MDD using mood disorder (including MDD, dysthymia, and bipolar disorder type 2) instead of MDD as a diagnostic category.

## 3. Results

### 3.1. Sample Characteristics

A total of 155 participants were included in the IREM trial. The mean age was 38.54 years (*SD* = 11.17), and 76.8% of the participants were female. The most frequent index traumas were sexual (58.7%) and physical abuse (20.0%). Most participants reported experiencing their trauma multiple times; only 6.8% in the IR condition and 21.0% in the EMDR condition reported a single traumatic event. The mean duration of PTSD symptoms was about 18 years, and 81.3% of the participants already had been in psychiatric or psychological treatment before. More details of recruitment and demographic characteristics can be found elsewhere [11,26]. Table 1 gives detailed information on comorbidity. A total of 127 (87.1%) of all participants had at least one comorbid psychiatric disorder; on average, participants had 2.22 (*SD* = 1.64) psychiatric comorbidities. The most common comorbidity was MDD, followed by AD (including OCD), summarized in one category to avoid multiple subgroups with low prevalence. The overall drop rate was 7.7%, 8.1% in the IR, and 7.4% in the EMDR condition. Eighteen participants (11.6%) completed treatment with less than 12 sessions (8.1% IR and 14.8% EMDR).

### 3.2. Primary Outcome

**Figure 1:**
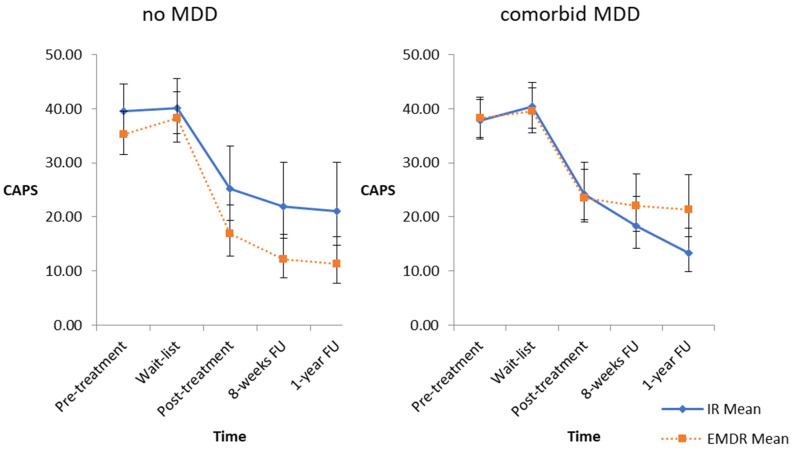
Course of posttraumatic stress symptoms in patients with and without major depressive disorder. Abbreviations: MDD = major depressive disorder; CAPS = clinician-administered PTSD scale for DSM-5; IR = imagery rescripting; EMDR = eye movement desensitization and reprocessing; FU = follow-up. Notes: Estimated CAPS means and 95-% confidence interval for all time points, treatment condition, and comorbid diagnosis of MDD.

The analysis of the primary outcome showed a significant three-way interaction (time, condition and MDD) (*F*_4, 109_ = 3.43, *p* = 0.012). Patients with comorbid MDD improved more from the pre-treatment to the eight-week follow-up assessment, respectively, the one-year follow-up assessment, if they were treated with IR. In contrast, patients without MDD showed a better improvement when treated with EMDR (Table 2 and Figure 1). Significant differences between pre-treatment and post-treatment (*t* = −9.01, *p* < 0.001), pre-treatment and eight-week follow up (*t* = −10.72, *p* < 0.001) as well as pre-treatment and one-year follow up (*t =* −10.56, *p* < 0.001) indicate a main effect for time. There was no significant interaction between MDD and time. 

No interactions could be found for comorbid AD, indicating that AD is neither a predictor nor a moderator to the treatment outcome (Table 3).

### 3.3. Secondary Outcomes

Analyses of the secondary outcome IES-R (index trauma) showed a three-way interaction between time, treatment condition, and diagnosis of MDD (*F*_4, 115_ = 2.62, *p* = 0.04), which has to be considered as not statistically significant due to Bonferroni correction for multiple testing. Nevertheless, a similar pattern as with the CAPS emerged (Supplementary Table S1). Significant differences between pre-treatment and post-treatment (*t* = −11.21, *p* < 0.001), pre-treatment and 8-weeks follow up (*t* = −10.80, *p* < 0.001) as well as pre-treatment and one-year follow up (*t* = −10.29, *p* < 0.001) indicate a main effect for time. There was no significant interaction between MDD and time. 

The secondary outcome IES-R (all traumas) showed a similar pattern as the primary outcome with a significant three-way interaction between time, treatment condition, and diagnosis of MDD (*F*_4, 115_ = 3.25, *p* = 0.015). Patients with comorbid MDD showed a better improvement from the pre-treatment to the one-year follow-up assessment (*t* = 3.03, *p* = 0.003) if they were treated with IR, whereas patients without MDD showed a better improvement if they were treated with EMDR. No significant interaction between time and MDD could be found. 

For AD using the IES-R (index trauma) and IES-R (all traumas) as outcome variables the GLMM showed no significant interactions and thereby the same pattern as the analysis of the primary outcome (Supplementary Table S1). Again significant differences between pre-treatment and post-treatment (IES-R index: *t* = −11.62, *p* < 0.001, IES-R all: *t* = −10.33, *p* < 0.001), pre-treatment and eight-week follow up (IES-R index: *t* = −11.19, *p* < 0.001; IES-R all: *t* = −10.72, *p* < 0.001) as well as pre-treatment and one-year follow up (IES-R index: *t* = −10.35, *p* < 0.001, IES-R all: *t* = −9.31, *p* < 0.001) indicate a main effect for time.

### 3.4. Sensitivity Analysis

The sensitivity analysis using mood disorder (MDD, dysthymia, and bipolar disorder type 2) as diagnostic category showed significant three-way interaction between time, treatment condition, and diagnosis of mood disorder (*F*_4, 110_ = 2.75, *p* = 0.03), but no significant interaction between time and mood disorder. Patients with comorbid mood disorder showed a greater reduction of symptoms from the pre-treatment to the one-year follow-up assessment if they were treated with IR. In contrast, patients without mood disorders improved more if they were treated with EMDR (Supplementary Table S2). Significant differences between pre-treatment and post-treatment (*t* = −8.04, *p* < 0.001), pre-treatment and eight-week follow up (*t* = −9.62, *p* < 0.001) as well as pre-treatment and one-year follow up (*t* = −9.48, *p* < 0.001) indicate a main effect for time. There was no significant interaction between mood disorder and time. 

## 4. Discussion

The purpose of this study was to examine comorbidities (MDD and AD) in PTSD as predictors of treatment outcome and as potential moderators with a differential impact on EMDR and IR. Contrary to our hypothesis we did not find that MDD or AD was related to a lower treatment outcome when the conditions were combined. We did find a significant differential effect of comorbid MDD on the PTSD outcome depending on the treatment condition: patients with comorbid MDD had a better outcome if they were treated with IR, whereas patients without MDD showed a better outcome if they were treated with EMDR. 

### 4.1. Effects of Major Depressive Disorder

Contrary to our results, a study on women with PTSD from sexual assault found that higher depression scores were associated with better improvement in PTSD symptomatology after treatment with cognitive processing therapy or prolonged exposure, but there were no differential effects of the two methods [8]. Matching our results, IR has shown potential as a stand-alone treatment for depressed patients with intrusive memories [37]. Our findings are also in line with a study that found a negative impact of comorbid MDD in refugees treated with EMDR [17]. However, in RCTs, EMDR has been shown to be effective as a treatment for depression when participants did not have comorbid PTSD [38,39]. 

Even though both treatment conditions reached satisfactory improvements in patients with MDD as well as in patients without MDD, the moderating effect might indicate different pathways of change. IR has been found to facilitate changing the meaning of traumatic events and, compared to traditional exposure, resulted in less self-blame [28]. In the second half of IR therapy in the current study, the patient imagined their adult-self returning to the trauma and provide for any unmet needs as a child. Thus, the reduction in self-blame and imagining a new positive self might produce particularly strong effects for PTSD patients with MDD as these latter sessions challenge the depressionogenic sense of self. Moreover, the focus on changing the patients’ emotional and interpersonal experience in IR [28] might be especially suitable for patients with MDD, whose formal-operational thinking might be restricted due to their depressive symptoms [40,41]. 

Cloitre et al. [9] also found that severity of depression moderated the outcome in treatment for female patients with PTSD related to childhood abuse. Patients with severe and moderate depression showed superior PTSD symptom reduction in a two-module-treatment that combined Skills Training in Affective and Interpersonal Regulation (STAIR) followed by a trauma-focused component, narrative therapy, vs. two control conditions (STAIR plus Supportive Counseling and Narrative Therapy plus Supportive Counseling). Among those with low levels of depressive symptoms, outcomes did not differ across the three treatment conditions. The authors conclude that for individuals with PTSD and comorbid depression a combined treatment approach in which skills training is added to trauma-focused work may be particularly beneficial [9]. Emotion regulation and coping skills are addressed in many PTSD approaches, however, most often this is not done as explicitly as in STAIR [24]. Additionally, in EMDR and IR emotion regulation and coping skills are not directly targeted, but improvements in this area might happen as a ‘byproduct’. Especially in IR patients receive explanations and validation of their emotions, alternative coping strategies are modeled in the first half of the treatment by the therapist entering the image and taking care of the patient’s child-self and finally, in the second half of therapy, new coping skills are trained by patients themselves by imaging themselves coping better. Thus, if improvement of emotion regulation and coping skills should turn out to be an essential component for PTSD patients with comorbid depression, this might explain why IR better met the needs of these patients as compared to EMDR in our study. However, in this study therapists were prevented in using imaginal techniques in the EMDR condition in order to ensure that there would be clear separation of the conditions. Such interventions are more routine when treating depression with EMDR [42]. The findings highlight the need to further assess the benefits of this therapeutic process in people presenting with comorbid depression and PTSD.

EMDR, on the other hand, was more efficient than IR in reducing PTSD symptoms in the absence of MDD. Meta-analytic data supports eye movements’ effect in reducing the target memory’s vividness and emotionality in clinical and laboratory studies [29]. In the IREM trial, patients in the EMDR condition had lower scores than those in IR after the first six sessions (mid-treatment) on measures of PTSD symptoms concerning the index trauma (but not all traumas) [11]. It might be that PTSD patients without MDD receiving EMDR were better able to notice this change as positive and thus become optimistic in dealing with other trauma memories. This explanation would be in line with not yet published results from the qualitative part of the study: Patients receiving IR reported a more positive sense of self, and those receiving EMDR said to be less bothered by their memories. Another explanation could involve the working memory theory as an underlying working mechanism of EMDR, which suggests that an external attention task (e.g., following the therapist’s finger with the eyes) taxes the capacity of the working memory and by this impedes recall of the trauma memory leading to changes in memory vividness and emotionality [30]. This mechanism might be impaired by cognitive symptoms of MDD—such as concentration problems and rumination—and might work more effectively in PTSD patients without comorbid MDD. 

Another possible explanation for the moderating effect of depression is that patients with comorbid MDD have different needs than those without comorbid MDD. Depression is a disorder in which (learned) helplessness plays a role as well as the paradoxical combination of social withdrawal and the need for social support and love. IR offers increased perceived control [43] as well as the experience of social support and understanding, perhaps making it better suitable for Ch-PTSD with comorbid depression. Another post-hoc hypothesis is that Ch-PTSD with depression is associated with a different trauma learning history than Ch-PTSD without depression, characterized by higher levels of emotional neglect, loneliness, and experiences of rejection. Such contextual experiences might strongly influence the dysfunctional meanings attached to the traumatic experiences that qualify for the A criterion of the PTSD diagnosis [44], and might be especially suitable to be addressed with IR. In contrast, Ch-PTSD without depression might be more characterized by intrusive experiences that hinder the patient, and that are especially suitable to be treated by EMDR. It is important that these post-hoc hypotheses are put to the test in future studies, as they help us to understand why depression moderates the effectiveness of IR vs. EMDR.

Conversely, a recent review summarizing the current evidence on the effects of EMDR for depression suggests that EMDR may be considered an effective treatment for improving symptoms of depression [45]. However, considering our findings’ tentative nature, these explanations are only hypothetical, and further studies are required to replicate our results and better understand the effects. 

### 4.2. Effect of AD

In our study, AD was neither a predictor nor a moderator of treatment outcome. This finding is in line with a study comparing EMDR with a combination of imaginal exposure and cognitive restructuring [20]. Also, in the study by van Minnen et al., general anxiety was not related to treatment outcome in patients with chronic PTSD treated using prolonged exposure [19]. On the other hand, AD is associated with lower remission rates of PTSD symptoms in epidemiological studies [13,14,15]. This difference might be explained by the fact that participants in treatment studies gave their commitment to treatment, meaning that they have decided to address their problems, to overcome avoidance and face trauma-focused work, while in individuals with AD in epidemiological studies, avoidance patterns might remain strong and change of PTSD symptoms is unlikely. 

Maybe we did not find any effect of AD because the AD sample was too heterogeneous, but the prevalence of separate ADs was too low to use them for our analysis. Based on our results, EMDR and IR can be recommended to PTSD patients with and without AD to the same degree. 

### 4.3. Limitations and Strengths

In the IREM study, patients with comorbid SUD were excluded, which is an important limitation for this study on comorbidities. It should also be noted that this was a sample of patients with Ch-PTSD. It has to be investigated whether the moderating effect of MDD can also be found in samples with different index traumas and PTSD with later onset. The IREM study limitations also apply to this study and are described elsewhere [11].

Strengths of the IREM trial were the high standard of treatment and data collection, the large international sample, the long-term follow-up, and the recruitment from regular health centers. The most important strength of this analysis was its contribution to the development of personalized treatment, i.e., improving treatment outcomes for individual PTSD patients by considering their comorbidities. 

## 5. Conclusions

To conclude, this study showed the importance of diagnosing comorbid MDD when offering patients with Ch-PTSD a particular treatment. Even though there was no difference between EMDR and IR in the primary analysis, this study provided recommendations for optimizing treatment for patients with and without MDD. However, a replication of these findings is warranted to put these on a more secure footing. In general, our results suggest that it is worth further exploring the effect of comorbid disorders on different PTSD treatments in more detail. EMDR and IR could be compared to established types of PTSD treatment like cognitive processing therapy, prolonged imaginal exposure, or PTSD-specific cognitive behavior therapy in these subgroups to strengthen the evidence and optimize treatment choice. Such information will help to better adapt treatments to the needs of PTSD patients with comorbid psychiatric disorders and to prepare the grounds for a personalized medicine approach to the treatment of PTSD.

## Figures and Tables

**Table 1:** Psychiatric comorbidities of the IREM sample.

	Treatment Condition, No. (%) of Patients		
	Total	IR	EMDR
Comorbid disorders	*n* = 155 (%)	*n* = 74 (%)	*n* = 81 (%)
Mood disorder	112 (72.3)	55 (74.3)	57 (70.4)
MDD	97 (62.6)	46 (62.2)	51 (63.0)
Dysthymia	17 (11)	7 (9.5)	10 (12.3)
Bipolar disorder type 2 ^1^	6 (3.9)	4 (5.4)	2 (2.5)
Anxiety disorder	87 (56.1)	38 (51.4)	49 (60.5)
Panic disorder	19 (12.3)	9 (12.2)	10 (12.3)
Panic with agoraphobia	26 (16.8)	11 (14.9)	15 (18.5)
Agoraphobia	5 (3.2)	1 (1.4)	4 (4.9)
Social phobia	37 (23.9)	17 (23.0)	20 (24.7)
OCD	20 (12.9)	9 (12.2)	11 (13.6)
GAD	28 (18.1)	12 (16.2)	16 (19.8)
Specific phobia	12 (7.7) ^2^	5 (6.8)	7 (8.6)
Eating disorder	21 (13.5)	8 (10.8)	13 (16.0)
Substance abuse	17 (11.0)	7 (9.5)	10 (12.3)
Other diagnosis	39 (25.2)	16 (21.6)	23 (28.4)

Abbreviations: MDD = Major depressive disorder; OCD = Obsessive compulsive disorder; MDD = MGAD = Generalized anxiety disorder. Notes: ^1^ biploar disorder type 1 was excluded, ^2^ no information about this diagnosis for 40 participants (25.8%).

**Table 2:** Primary treatment outcome CAPS-5 by MDD diagnosis and treatment condition across all assessment points.

Timepoint	MDD No						MDD Yes						Time by MDD		
	*N*			Estimated Means (95% CI)		d ^a^	*N*			Estimated Means (95% CI)		d ^a^	t ^b^	df ^b^	p ^b^
Wait-list	32			39.18 (35.49–43.24)		0.12	56			39.98 (36.55–43.73)		0.13	0.08	106	0.94
Pre-treatment	55			37.40 (34.11–41.00)			92			38.04 (35.06–41.28)					
Post-treatment	49			20.65 (16.98–25.12)		1.55	82			23.84 (20.45–27.78)		1.22	1.22	106	0.23
Eight-week follow-up	47			16.31 (12.99–20.49)		2.16	79			20.13 (16.85–24.04)		1.66	1.54	106	0.13
One-year follow-up	38			15.41 (11.90–19.97)		2.31	67			16.87 (13.82–20.61)		2.12	0.49	106	0.63
	**IR**			**EMDR**			**IR**			**EMDR**			**Time by Treatment by MDD**		
	**Estimated Means**			**Estimated Means**			**Estimated Means**			**Estimated Means**			**t ^b^**	**df ^b^**	**p ^b^**
	***N***	**(95% CI)**	**d ^a^**	***N***	**(95% CI)**	**d ^a^**	***N***	**(95% CI)**	**d ^a^**	***N***	**(95% CI)**	**d ^a^**			
Wait-list	15	40.13 (35.35–45.56)	0.04	17	38.24 (33.85–43.19)	0.21	24	40.41 (36.39–44.89)	0.17	32	39.55 (35.61–43.92)	0.09	−1.14	109	0.26
Pre-treatment	26	39.59 (35.15–44.60)		29	35.32 (31.53–39.58)		44	37.88 (34.39–41.72)		48	38.20 (34.69–42.07)				
Post-treatment	26	25.26 (19.31–33.05)	1.17	23	16.88 (12.80–22.27)	1.93	38	24.19 (19.46–30.06)	1.17	44	23.49 (19.11–28.88)	1.27	1.22	109	0.22
Eight-week follow-up	23	21.93 (16.41–27.81)	1.54	24	12.14 (8.79–16.75)	2.79	36	18.38 (14.23–23.74)	1.89	43	22.04 (17.37–27.94)	1.43	2.62	109	0.01
One-year follow-up	19	21.09 (14.75–30.16)	1.64	19	11.26 (7.79–16.27)	2.98	30	13.33 (9.94-17.88)	2.72	37	21.36 (16.41–27.81)	1.51	3.26	109	0.001

Abbreviations: CAPS-5 = clinical administered PTSD scale for DSM-5; MDD = major depressive disorder; IR = imagery rescripting; EMDR = eye movement desensitization and reprocessing. Notes: analyses by generalized linear mixed models (GLMM), using a negative binomial distribution with a loglink to deal with the skewed distribution of the dependent variable, an unstructured covariance structure for the repeated part, and a random effect of time at the level of site (see [22]). ^a^ Effect size calculated for treatment condition compared to pre-treatment, based on estimated means in the transformed scale and the baseline standard deviation derived from the pre-treatment variance estimated with a GLMM analysis with only an unstructured repeated part and a fixed intercept [11]. Because of unequal sample sizes in the four groups (MDD and IR, MDD and EMDR, no MDD and IR, no MDD and EMDR), the average effect sizes are higher than the primary analysis [10]. ^b^ differences between treatments and diagnosis of MDD in change from pre-treatment based on a repeated GLMM using centered, dimensional predictors to interpret lower-level interactions.

**Table 3:** Primary treatment outcome CAPS-5 by AD diagnosis and treatment condition across all assessment points.

Timepoint	AD No						AD Yes						Time by AD		
	*N*			Estimated Means (95% CI)		d ^a^	*N*			Estimated Means (95% CI)		d ^a^	t ^b^	df ^b^	p^b^
Wait-list	32			39.89 (35.87–44.36)		0.20	56			39.74 (35.74–44.18)		0.03	−1.51	144	0.14
Pre-treatment	63			36.73 (33.28–40.53)			84			39.27 (35.60–43.32)					
Post-treatment	58			21.84 (18.13–26.30)		1.36	73			23.28 (19.61–27.63)		1.36	−0.03	144	0.98
Eight-week follow-up	55			19.52 (15.82–24.09)		1.65	71			18.37 (15.13–22.30)		1.98	−1.06	144	0.29
One-year follow-up	50			17.55 (13.84–22.26)		1.93	55			17.00 (13.56–21.31)		2.18	−0.67	144	0.51
	**IR**			**EMDR**			**IR**			**EMDR**		**Time by Treatment by AD**			
	**Estimated Means**			**Estimated Means**			**Estimated Means**			**Estimated Means**			**t ^b^**	**df ^b^**	**p ^b^**
	***N***	**(95% CI)**	**d ^a^**	***N***	**(95% CI)**	**d ^a^**	***N***	**(95% CI)**	**d ^a^**	***N***	**(95% CI)**	**d ^a^**			
Wait-list	15	38.93 (34.20–44.32)	0.13	17	40.88 (35.98–46.43)	0.31	24	41.27 (36.59–46.55)	0.07	32	38.26 (34.00–43.06)	0.01	−1.23	108	0.22
Pre-treatment	33	37.13 (33.09–41.67)		30	36.33 (32.25–40.93)		37	40.17 (35.83–45.04)		47	38.39 (34.44–42.79)				
Post-treatment	34	24.50 (19.29–31.12)	1.08	24	19.46 (14.84–25.52)	1.63	30	24.51 (19.13–31.39)	1.29	43	22.11 (17.83–27.43)	1.44	0.75	108	0.46
Eight-week follow-up	29	20.95 (15.85–27.68)	1.49	26	18.19 (13.44–24.62)	1.80	30	18.28 (13.78–24.25)	2.05	41	18.46 (14.44–23.60)	1.91	0.75	108	0.46
One-year follow-up	28	15.83 (11.55–21.70)	2.22	22	19.46 (13.80–27.44)	1.63	21	17.12 (12.19–24.04)	2.22	34	16.88 (12.73–22.38)	2.14	−0.68	108	0.50

Abbreviations: CAPS-5 = clinical administered PTSD scale for DSM-5; AD = anxiety disorder; IR = imagery rescripting; EMDR = eye movement desensitization and reprocessing. Notes: analyses by generalized linear mixed models (GLMM), using a negative binomial distribution with a loglink to deal with the skewed distribution of the dependent variable, an unstructured covariance structure for the repeated part, and a random effect of time at the level of site (see [22]). ^a^ Effect size calculated for treatment condition compared to pre-treatment, based on estimated means in the transformed scale and the baseline standard deviation derived from the pre-treatment variance estimated with a GLMM analysis with only an unstructured repeated part and a fixed intercept [11]. Because of unequal sample sizes in the four groups (AD and IR, AD and EMDR, no AD and IR, no AD and EMDR), the average effect sizes are higher than the primary analysis [10]. ^b^ differences between treatments and diagnosis of AD in change from pre-treatment based on a repeated GLMM using centered, dimensional predictors to interpret lower-level interactions.

## Data Availability

The data that support the findings of this study are available on request from the corresponding author, N.A. The data are not publicly available because they contain information that could compromise the privacy of research participants.

## Supplementary Materials

The following are available online at https://www.mdpi.com/article/10.3390/jcm10163708/s1, Table S1. Secondary treatment outcomes by MDD and AD diagnosis and treatment condition across all assessment points, Table S2. Primary outcome CAPS-5 by mood disorder (MD) diagnosis and treatment condition across all assessment points.
