# Supplementary material for: Differential Effects of Comorbid Psychiatric Disorders on Treatment Outcome in Posttraumatic Stress Disorder from Childhood Trauma
Source: J Clin Med. 2021 Aug 20;10(16):3708. doi: 10.3390/jcm10163708 (PMC8397108; doi:10.3390/jcm10163708)
Supplement: Supplementary file 1 [file jcm-10-03708-s001.zip › IREMComorbidity_Supplementary Materials.pdf]

Supplementary Table S1. Secondary treatment outcomes by MDD and AD diagnosis and treatment condition across all assessment points.

Outcome									
**IES-R index trauma**	**MDD no**			**MDD yes**			Time by MDD		
Timepoint	N	Estimated Means (95% CI)	d^a	N	Estimated Means (95% CI)	d^a	t^b	df^b	p^b
Wait-list	33	52.13 (48.27 – 56.30)	0.19	59	53.58 (50.53 – 56.83)	0.21	0.17	115	.87
Pre-treatment	57	48.88 (44.99 – 53.10)		95	49.91 (46.81 – 53.21)				
Post-treatment	53	18.06 (13.65 – 23.88)	2.96	86	21.37 (17.18 – 26.58)	2.52	0.90	115	.37
8-week Follow-up	47	15.50 (11.35 – 21.17)	3.41	80	21.50 (16.91 – 27.34)	2.50	1.66	115	.10
1-year Follow-up	38	17.20 (12.24 – 24.15)	3.10	68	17.95 (13.82 – 23.32)	3.04	0.11	115	.91

	**IR**			**EMDR**			**IR**			**EMDR**			Time by treatment by MDD		
	N	Estimated Means (95% CI)	d^a	N	Estimated Means (95% CI)	d^a	N	Estimated Means (95% CI)	d^a	N	Estimated Means (95% CI)	d^a	t^b	df^b	p^b
Wait-list	15	54.47 (48.70 – 60.93)	0.15	18	49.89 (44.89 - 55.44)	0.23	25	53.16 (48.72 – 58.01)	0.25	34	54.01 (49.92 – 58.43)	0.17	-0.61	115	.54
Pre-treatment	28	51.71 (45.93 – 58.23)		29	46.20 (41.15 – 51.85)		45	48.93 (44.58 – 53.70)		50	50.91 (46.61 – 55.60)				
Post-treatment	27	24.62 (16.65 – 36.42)	2.21	26	13.24 (8.88 – 19.73)	3.72	40	22.28 (16.22 – 30.59)	2.34	46	20.50 (15.20 – 27.66)	2.70	1.17	115	.24
8-week Follow-up	22	23.80 (15.36 – 36.89)	2.31	25	10.10 (6.48 – 15.72)	4.52	37	19.17 (13.49 – 27.23)	2.79	43	24.12 (17.38 – 33.47)	2.22	2.54	115	.01
1-year Follow-up	19	25.45 (15.81 – 40.97)	2.11	19	11.62 (7.16 – 18.86)	4.10	30	14.52 (9.86 – 21.39)	3.61	38	22.19 (15.61 – 31.56)	2.47	2.63	115	.01

Outcome									
**IES-R all traumas**		**MDD no**			**MDD yes**		Time by MDD		
Timepoint	N	Estimated Means (95% CI)	d^a	N	Estimated Means (95% CI)	d^a	t^b	df^b	p^b
Wait-list	33	45.31 (41.01 – 50.06)	0.07	59	48.19 (44.65 – 52.02)	0.02	-0.50	115	.62
Pre-treatment	57	43.90 (39.73 – 48.51)		94	47.69 (44.15 – 51.53)				
Post-treatment	53	21.90 (17.03 – 28.18)	1.47	86	23.66 (19.43 – 28.80)	1.48	-0.04	115	.97
8-week Follow-up	47	18.72 (14.31 – 24.48)	1.80	80	23.38 (19.00 – 28.77)	1.51	0.94	115	.35
1-year Follow-up	38	20.30 (14.93 – 27.62)	1.63	67	19.90 (15.69 – 25.25)	1.85	-0.59	115	.56

	**IR**			**EMDR**			**IR**			**EMDR**			Time by treatment by MDD		
	N	Estimated Means (95% CI)	d^a	N	Estimated Means (95% CI)	d^a	N	Estimated Means (95% CI)	d^a	N	Estimated Means (95% CI)	d^a	t^b	df^b	p^b
Wait-list	15	50.87 (44.02 – 58.78)	0.12	18	40.36 (35.18 – 46.30)	0.02	25	46.57 (41.60 – 52.13)	0.04	34	49.87 (44.99 – 55.29)	0.01	0.40	115	.69
Pre-treatment	28	48.11 (41.69 – 55.51)		29	40.06 (34.85 – 46.05)		44	45.75 (40.89 – 51.19)		50	49.72 (44.71 – 55.29)				
Post-treatment	27	26.70 (18.72 – 38.07)	1.24	26	17.97 (12.57 – 25.69)	1.69	40	22.97 (17.25 – 30.59)	1.46	46	24.37 (18.61 – 31.91)	1.51	0.69	115	.50
8-week Follow-up	22	25.80 (17.63 – 37.74)	1.32	25	13.58 (9.30 – 19.83)	2.28	37	21.29 (15.72 – 28.82)	1.62	43	25.68 (19.34 – 34.10)	1.39	1.89	115	.06
1-year Follow-up	19	28.09 (18.21 – 43.32)	1.14	19	14.68 (9.48 – 22.72)	2.12	29	14.23 (9.98 – 20.29)	2.47	38	27.83 (20.27 – 38.22)	1.23	3.03	115	.003

IES-R index trauma	AD no			AD yes			Time by AD		
Timepoint	N	Estimated Means (95% CI)	d[a]	N	Estimated Means (95% CI)	d[a]	t[b]	df[b]	p[b]
Wait-list	36	50.11 (46.67 – 53.81)	0.19	56	55.15 (51.91 – 58.60)	0.21	0.24	118	.81
Pre-treatment	66	47.06 (43.59 – 50.79)		86	51.31 (47.95 – 54.90)				
Post-treatment	63	(17.25 (13.40 – 22.20)	2.98	76	22.13 (17.62 – 27.80)	2.50	1.03	118	.31
8-week Follow-up	57	18.19 (13.98 – 23.67)	2.83	70	20.65 (16.26 – 26.22)	2.71	0.24	118	.81
1-year Follow-up	52	17.18 (12.80 – 23.06)	2.99	54	20.06 (15.20 – 26.49)	2.79	0.37	118	.72

	IR			EMDR			IR			EMDR			Time by treatment by AD		
	N	Estimated Means (95% CI)	d[a]	N	Estimated Means (95% CI)	d[a]	N	Estimated Means (95% CI)	d[a]	N	Estimated Means (95% CI)	d[a]	t[b]	df[b]	p[b]
Wait-list	17	51.86 (46.90 – 57.35)	0.16	19	48.42 (43.77 – 53.56)	0.21	23	55.30 (50.44 – 60.63)	0.26	33	55.01 (50.83 – 59.52)	0.18	-0.10	118	.39
Pre-treatment	36	49.14 (44.28 – 54.53)		30	45.06 (40.29 – 50.39)		37	50.78 (45.87 – 56.22)		49	51.84 (47.41 – 56.67)				
Post-treatment	36	22.83 (16.39 – 31.82)	2.28	27	13.03 (8.90 – 19.07)	3.69	31	23.49 (16.59 – 33.27)	2.29	45	20.85 (15.52 – 28.01)	2.71	-1.94	118	.06
8-week Follow-up	30	21.94 (15.43 – 31.20)	2.40	27	15.07 (10.19 – 22.30)	3.26	29	20.08 (13.94 – 28.94)	2.76	41	21.22 (15.58 – 28.90)	2.66	-0.77	118	.45
1-year Follow-up	29	16.80 (11.33 – 24.93)	3.19	23	17.56 (11.34 – 27.18)	2.80	20	21.91 (14.25 – 33.69)	2.50	34	18.37 (12.92 – 26.11)	3.08	-0.18	118	.86

**IES-R all traumas**	**AD no**			**AD yes**			Time by AD		
Timepoint	N	Estimated Means (95% CI)	d[a]	N	Estimated Means (95% CI)	d[a]	t[b]	df[b]	p[b]
Wait-list	36	45.29 (41.30 – 49.66)	0.02	56	48.54 (44.85 – 52.54)	0.05	0.40	114	.69
Pre-treatment	65	44.90 (41.00 – 49.18)		86	47.33 (43.67 – 51.30)				
Post-treatment	63	22.66 (17.96 – 28.60)	1.44	76	23.16 (18.75 – 28.60)	1.51	-0.23	114	.82
8-week Follow-up	57	21.52 (16.90 – 27.40)	1.55	70	21.51 (17.27 – 26.78)	1.67	-0.37	114	.71
1-year Follow-up	51	21.47 (16.33 – 28.23)	1.56	54	20.87 (16.10 – 27.06)	1.73	-0.49	114	.63

	**IR**			**EMDR**			**IR**			**EMDR**			Time by treatment by AD		
	N	Estimated Means (95% CI)	d[a]	N	Estimated Means (95% CI)	d[a]	N	Estimated Means (95% CI)	d[a]	N	Estimated Means (95% CI)	d[a]	t[b]	df[b]	p[b]
Wait-list	17	47.71 (41.92 – 54.30)	0.05	19	42.99 (37.70 – 49.02)	0.01	23	48.64 (43.15 – 54.83)	0.09	33	48.45 (43.69 – 53.72)	0.01	-0.09		.93
Pre-treatment	35	46.66 (41.20 – 52.84)		30	43.21 (37.84 – 49.35)		37	46.60 (41.29 – 52.60)		49	48.08 (43.24 – 53.47)				
Post-treatment	36	25.72 (18.88 – 35.05)	1.26	27	19.97 (14.10 – 28.27)	1.63	31	23.02 (16.69 – 31.76)	1.49	45	23.29 (17.72 – 30.62)	1.53	0.58		.56
8-week Follow-up	30	26.15 (18.90 – 36.18)	1.22	27	17.70 (12.38 – 25.32)	1.88	29	19.75 (14.14 – 27.60)	1.81	41	23.42 (17.63 – 31.10)	1.52	1.59		.11
1-year Follow-up	28	20.73 (14.34 – 29.98)	1.71	23	22.24 (14.85 – 33.32)	1.40	20	18.50 (12.36 – 27.71)	1.95	34	23.54 (16.99 – 32.62)	1.51	0.19		.85

Abbreviations: IES-R, Impact of Event Scale-Revised; MDD, Major Depressive Disorder; AD, Anxiety Disorder; IR, Imagery Rescripting; EMDR, Eye Movement Desensitization and Reprocessing.

Note. Analyses by Generalized Linear Mixed Models (GLMM), using a Negative Binomial Distribution with a loglink to deal with the skewed distribution of the dependent variable, an unstructured covariance structure for the repeated part, and a random effect of time at the level of site (see [22]).

[a]Effect size calculated for treatment condition compared to pre-treatment, based on estimated means in the transformed scale and the baseline standard deviation derived from the pre-treatment variance estimated with a GLMM analysis with only an unstructured repeated part and a fixed intercept [11]. Because of unequal sample sizes in the four groups (MDD/AD and IR, MDD/AD and EMDR, no MDD/AD and IR, no MDD/AD and EMDR), the average effect sizes are higher than the primary analysis [10].

[b]differences between treatments and diagnosis of MDD/AD in change from pre-treatment based on a repeated GLMM using centered, dimensional predictors to interpret lower-level interactions.

Supplementary Table S2. Primary outcome CAPS-5 by Mood Disorder (MD) diagnosis and treatment condition across all assessment points.

Timepoint	**MD no**			**MD yes**			Time by MD		
	N	Estimated Means (95% CI)	d[a]	N	Estimated Means (95% CI)	d[a]	t[b]	df[b]	p[b]
Wait-list	21	38.74 (34.50 – 43.50)	0.08	67	40.22 (36.57 – 44.24)	0.15	0.50	100	.62
Pre-treatment	40	37.62 (33.79 – 41.88)		107	38.02 (34.87 – 41.47)				
Post-treatment	38	21.67 (17.30 – 27.14)	1.44	93	23.21 (20.03 – 26.89)	1.29	0.52	100	.61
8-week Follow-up	36	17.94 (13.85 – 23.23)	1.93	90	19.36 (16.38 – 22.88)	1.76	0.50	100	.62
1-year Follow-up	30	17.50 (13.11 – 23.37)	2.00	75	16.18 (13.42 – 19.51)	2.23	-0.57	100	.57

	**IR**			**EMDR**			**IR**			**EMDR**			Time by treatment by MD		
	N	Estimated Means (95% CI)	d[a]	N	Estimated Means (95% CI)	d[a]	N	Estimated Means (95% CI)	d[a]	N	Estimated Means (95% CI)	d[a]	t[b]	df[b]	p[b]
Wait-list	9	40.04 (34.20 – 46.87)	0.05	12	37.48 (32.57 – 43.14)	0.10	30	40.43 (36.43 – 44.88)	0.14	37	40.01 (35.93 – 44.56)	0.15	-0.18	110	.85
Pre-treatment	17	39.30 (33.99 – 45.45)		23	36.01 (31.65 – 40.97)		53	38.30 (34.78 – 42.18)		54	37.75 (34.19 – 41.69)				
Post-treatment	18	24.21 (17.52 – 33.46)	1.26	20	19.40 (14.35 – 26.23)	1.61	46	24.71 (20.22 – 30.18)	1.14	47	21.81 (17.84 – 26.65)	1.43	0.11	110	.91
8-week Follow-up	16	22.11 (15.22 – 32.13)	1.50	20	14.55 (10.28 – 20.60)	2.36	43	18.71 (14.83 – 23.60)	1.87	47	20.03 (15.97 – 25.13)	1.65	1.60	110	.11
1-year Follow-up	14	23.48 (15.45 – 35.38)	1.35	16	13.10 (8.84 – 19.43)	2.64	35	13.44 (10.29 – 17.55)	2.73	40	19.48 (15.16 – 25.04)	1.72	2.83	110	.006

Abbreviations: CAPS-5, Clinical Administered PTSD Scale for DSM-5; MD, Mood Disorder; IR, Imagery Rescripting; EMDR, Eye Movement Desensitization and Reprocessing.

Note. Analyses by Generalized Linear Mixed Models (GLMM), using a Negative Binomial Distribution with a loglink to deal with the skewed distribution of the dependent variable, an unstructured covariance structure for the repeated part, and a random effect of time at the level of site (see [22]).

[a]Effect size calculated for treatment condition compared to pre-treatment, based on estimated means in the transformed scale and the baseline standard deviation derived from the pre-treatment variance estimated with a GLMM analysis with only an unstructured repeated part and a fixed intercept [11]. Because of unequal sample sizes in the four groups (MD and IR, MD and EMDR, no MD and IR, no MD and EMDR), the average effect sizes are higher than the primary analysis [10].

[b]differences between treatments and MD diagnosis change from pre-treatment based on a repeated GLMM using centered, dimensional predictors to interpret lower-level interactions.
